# Supplementary material for: From sequence to enzyme mechanism using multi-label machine learning
Source: BMC Bioinformatics. 2014 May 19;15:150. doi: 10.1186/1471-2105-15-150 (PMC4229970; doi:10.1186/1471-2105-15-150)
Supplement: Additional file 2 — Java code of ml2db. Additional file ml2db_code.tar.gz contains the Java source code to run the multi-label machine learning experiments and save the results to database. The code’s Javadoc is included. [file 1471-2105-15-150-S2.zip › additional file 2/ml2db/ml2db/doc/mulan/classifier/neural/NormalizationFilter.html]

NormalizationFilter


JavaScript is disabled on your browser.


- Overview
- Package
- Class
- Use
- Tree
- Deprecated
- Index
- Help

- Prev Class
- Next Class

- Frames
- No Frames

- All Classes

- Summary:
- Nested |
- Field |
- Constr |
- Method

- Detail:
- Field |
- Constr |
- Method


mulan.classifier.neural

## Class NormalizationFilter

- java.lang.Object
- - mulan.classifier.neural.NormalizationFilter

- All Implemented Interfaces:
  :   java.io.Serializable

  ---

    

  ```
  public class NormalizationFilter
  extends java.lang.Object
  implements java.io.Serializable
  ```

  Performs a normalization of numeric attributes of the data set.
  It is initialized based on given `MultiLabelInstances` data set and then can
  be used to normalize `Instance` instances which conform to the format of the
  data set the `NormalizationFilter` was initialized from.

  Version:
  :   2012.02.27

  Author:
  :   Jozef Vilcek

  See Also:
  :   Serialized Form

- - ### Constructor Summary

    Constructors

    Constructor and Description

    `NormalizationFilter(MultiLabelInstances mlData,
    boolean performNormalization)`

    Creates a new instance of `NormalizationFilter` class for given data set.

    `NormalizationFilter(MultiLabelInstances mlData,
    boolean performNormalization,
    double minValue,
    double maxValue)`

    Creates a new instance of `NormalizationFilter` class for given data set.
  - ### Method Summary

    Methods

    | Modifier and Type | Method and Description |
    | --- | --- |
    | `void` | `normalize(weka.core.Instance instance)` Performs a normalization of numerical attributes on given instance. |

    - ### Methods inherited from class java.lang.Object

      `equals, getClass, hashCode, notify, notifyAll, toString, wait, wait, wait`

- - ### Constructor Detail


    - #### NormalizationFilter

      ```
      public NormalizationFilter(MultiLabelInstances mlData,
                         boolean performNormalization,
                         double minValue,
                         double maxValue)
      ```

      Creates a new instance of `NormalizationFilter` class for given data set.

      Parameters:
      :   `mlData` - the `MultiLabelInstances` data set from which normalizer
          should be initialized.
      :   `performNormalization` - indicates whether normalization of instances contained
          in the data set used for initialization should be performed
      :   `minValue` - the minimum value of the normalization range for numerical attributes
      :   `maxValue` - the maximum value of the normalization range for numerical attributes


    - #### NormalizationFilter

      ```
      public NormalizationFilter(MultiLabelInstances mlData,
                         boolean performNormalization)
      ```

      Creates a new instance of `NormalizationFilter` class for given data set.
      The normalizer will be initialized to perform normalization to the default
      range <-1,1>.

      Parameters:
      :   `mlData` - the `MultiLabelInstances` data set from which normalizer
          should be initialized.
      :   `performNormalization` - indicates whether normalization of instances contained
          in the data set used for initialization should be performed
  - ### Method Detail


    - #### normalize

      ```
      public void normalize(weka.core.Instance instance)
      ```

      Performs a normalization of numerical attributes on given instance.
      The instance must conform to format of instances data the `NormalizationFilter`
      was initialized with.

      Parameters:
      :   `instance` - the instance to be normalized


- Overview
- Package
- Class
- Use
- Tree
- Deprecated
- Index
- Help

- Prev Class
- Next Class

- Frames
- No Frames

- All Classes

- Summary:
- Nested |
- Field |
- Constr |
- Method

- Detail:
- Field |
- Constr |
- Method
